# The Association Between Thromboembolic Events and *ALK* , *ROS1* , *RET* Rearrangements or *EGFR* Mutations in Patients With Advanced Lung Adenocarcinoma: A Retrospective Cohort Study

**DOI:** 10.1111/1759-7714.70141

**Published:** 2025-08-02

**Authors:** Xiaohan Qian, Mengjiao Fu, Jing Zheng, Junjun Chen, Cuihong Cai, Jianya Zhou, Jianying Zhou

**Affiliations:** ^1^ Department of Respiratory Disease Thoracic Disease Center, the First Affiliated Hospital, Zhejiang University School of Medicine Hangzhou China; ^2^ State Key Laboratory for Diagnosis and Treatment of Infectious Diseases, National Clinical Research Centre for Infectious Diseases, Collaborative Innovation Centre for Diagnosis and Treatment of Infectious Diseases The First Affiliated Hospital, Zhejiang University School of Medicine Hangzhou China

**Keywords:** *ALK*, arterial thromboembolism, lung adenocarcinoma, *RET*, *ROS1*, venous thromboembolism

## Abstract

**Introduction:**

Previous studies have reported inconsistent findings regarding the associationbetween *ALK* and *ROS1* rearrangements in lung cancer and thromboembolic risk. This retrospective study aimed to investigate this association in advanced lung adenocarcinoma patients with *ALK*, *ROS1*, *RET* rearrangements, and *EGFR* mutations.

**Materials and Methods:**

We retrospectively collected information on patients with advanced lung adenocarcinoma in the First Affiliated Hospital of Zhejiang University School of Medicine from January 2013 to March 2021. All patients with confirmed *ALK*, *ROS1*, or *RET* rearrangements, as well as a comparison cohort of those with *EGFR* mutation, were included. Clinical characteristics were analyzed, and the association between driver genes and TE risks was analyzed using competing risk and logistic regression.

**Results:**

A total of 546 patients were included in the study. Among them, those with *ROS1* rearrangements exhibited the highest cumulative incidence of thromboembolic events (TEs), reaching 17.5% ± 0.2% during the peri‐diagnostic period (within 6 months following diagnosis). Regardless of the entire follow‐up or the peri‐diagnostic period, *ROS1* rearrangements were significantly associated with an increased risk of TEs. Multivariate analysis revealed *ROS1* rearrangements, the number of comorbidities, the size of mediastinal lymph nodes, and elevated C‐reactive protein (CRP) levels as TE risk factors during the peri‐diagnostic period. Throughout the follow‐up period, *ROS1* rearrangements and hypertension were independent TE risk factors. In addition, the development of TE significantly affected the overall survival of patients with *EGFR* mutations.

**Conclusion:**

*ROS1* rearrangements were significantly associated with an increased risk of TE.

## Graphical abstract

In this retrospective study of 546 lung adenocarcinoma patients with four different driver genes, *ROS1* rearrangements were associated with the increased risk of thromboembolic events (TEs), particularly during the peri‐diagnostic period (6 months before and after diagnosis). Risk factors of TEs during the peri‐diagnostic period included *ROS1* rearrangements, multiple comorbidities, enlarged mediastinal lymph nodes, and elevated CRP levels.

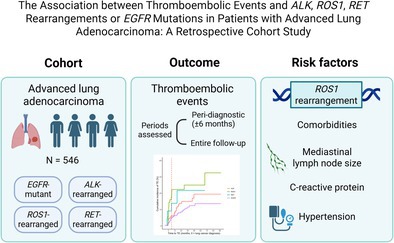

**Abbreviations**
ARMS‐PCR: amplification refractory mutation system–polymerase chain reaction
ATE: arterial thromboembolism
BMI: body mass index
CEA: carcinoembryonic antigen
COMPASS‐CAT: Computerized Registry of Patients with Solid Tumors for the Assessment of Venous Thromboembolism in Cancer—Clinical Assessment Tool
CRP: C‐reactive protein
DVT: deep vein thrombosis
ECOG: eastern cooperative oncology group performance status
FISH: fluorescence in situ hybridization
HR: hazard ratio
IHC: immunohistochemistry
IQR: inter‐quartile range
NA: Not applicable
NGS: next‐generation sequencing
NSCLC: non‐small‐cell lung cancer
OR: odds ratio
OS: overall survival
PE: pulmonary embolism
RECIST: response evaluation criteria in solid tumors
TE: thromboembolic event
TKI: tyrosine kinase inhibitor
VIF: variance inflation factor
VTE: venous thromboembolism

## 1 Introduction

Thromboembolic events (TEs), encompassing venous thromboembolism (VTE), arterial thromboembolism (ATE) and other related occurrences, are common complications in patients diagnosed with lung cancer [1]. VTE, which includes pulmonary embolism (PE), deep vein thrombosis (DVT) and venous thrombosis at other sites, is a major cause of morbidity and mortality in lung cancer [2, 3]. Compared with cancer patients without VTE, those with VTE face a 3‐ to 5‐fold heightened risk of mortality [4]. Notably, lung cancer has a comparatively elevated risk of VTE compared with other malignant tumors, with an incidence ranging from 7% to 13% [2, 5]. Furthermore, the incidence of thrombosis in adenocarcinoma is three to four times higher than that in squamous cell carcinoma and small‐cell lung cancer [6, 7]. Additionally, lung cancer seems to be associated with an increased risk of ATE, with an incidence ranging from 2% to 9% [8, 9].

Currently, some studies have reported an association between lung cancers with *ALK* and *ROS1* rearrangements and an elevated risk of TEs [10]. However, the results remain controversial [11]. The highest reported incidence of TEs in patients with *ALK* rearrangements comes from small real‐world cohorts (47%, *N* = 17; 42%, *N* = 55) [12, 13]. Nevertheless, the incidence of TEs significantly decreases in larger real‐world cohorts (17%, *N* = 70; 22%, *N* = 193) [11, 14]. In cohorts from clinical trials, the reduction was even more pronounced (1%–6%) in patients with *ALK* fusions [15, 16, 17, 18]. Regarding *ROS1* fusions, which share homology with *ALK* fusions [19, 20], several studies have indicated that patients with *ROS1* fusions have a significantly increased risk of TEs [12, 14, 21, 22, 23]. Similarly, prospective randomized controlled clinical trials of ROS1‐TKI did not demonstrate a higher incidence of TEs [15, 16, 18, 24]. *ALK*, *ROS1*, and *RET* are the most frequent recurring kinase fusions identified [25], all belonging to the receptor tyrosine kinase fusion family [26]. It has been reported that patients with *RET* fusions exhibit similarities to those with *ALK* and *ROS1* fusions in terms of histological characteristics and metastasis patterns [27, 28, 29]. A retrospective analysis found that the incidence of TEs in patients with *RET* fusions was as high as 48% [30], suggesting that, like *ALK* and *ROS1*, *RET* may also be associated with an increased risk of thromboembolism. However, due to the rarity of *RET* fusions, further evidence is needed to establish a robust correlation between *RET* fusions and the elevated risks of thromboembolism.

We conducted a retrospective study of patients with advanced lung adenocarcinoma harboring *ALK*, *ROS1*, and *RET* fusions, comparing them with cohorts harboring *EGFR* mutations. This study aimed to determine whether *ALK*, *ROS1*, and *RET* rearrangements are associated with increased thromboembolic risk compared to *EGFR* mutations, particularly during the peri‐diagnostic period (within 6 months before and after diagnosis). Additionally, we assessed TE risk over the entire follow‐up period, examined the influence of clinical features associated with driver gene status on TE occurrence, and analyzed the potential impact of TEs on overall survival (OS).

## 2 Methods

### 2.1 Study Population

Patients diagnosed with unresectable, locally advanced, or metastatic *ALK*+, *ROS1*+, and *RET*+ lung adenocarcinoma were identified through a comprehensive search of electronic medical records from January 2013 to March 2021 at the First Affiliated Hospital of Zhejiang University School of Medicine. As a control cohort, patients with *EGFR* mutations were selected by random stratification of TNM staging at a ratio of 1:1. The cohort with *EGFR* mutation was chosen as the control cohort to minimize confounding factors. Given the diverse expression of driver genes commonly observed in lung adenocarcinoma populations, selecting an *EGFR*+ cohort with its homogeneous genetic profile helps avoid the variability introduced by other driver genes. Individuals with other driver genes (e.g., *KRAS, BRAF, MET* exon 14 skipping) and those with less than 30 days of follow‐up were excluded from the analysis. *EGFR* mutations were identified by ARMS‐PCR (amplification refractory mutation system–polymerase chain reaction) or next‐generation sequencing (NGS), while *ALK*, *ROS1*, and *RET* rearrangements were detected by fluorescence in situ hybridization (FISH), immunohistochemistry (IHC) and/or NGS. All molecular tests were performed in the hospital's central laboratory following standardized protocols.

### 2.2 Patient Demographics and Risk Factors for TEs

Data on clinical characteristics, treatment response, and TEs were extracted from electronic medical records. TEs encompass VTE and ATE, with VTE including PE and DVT, and ATE including myocardial infarction and cerebrovascular events. Patients with clinical symptoms underwent imaging studies to confirm the presence of TEs. All incidentally detected TE cases were included in this study and reviewed by a panel of vascular and radiology experts. The last follow‐up date and survival status (alive or deceased) of each participant were recorded for survival analysis.

Other clinical and laboratory variables potentially influencing TE risks were categorized for univariate and multivariable analyses to retain statistical power and facilitate data collection. Among these, the Khorana risk score and the ONKOTEV score were included as key variables. The Khorana risk score, widely validated for assessing VTE risk in patients with cancer receiving chemotherapy [31], comprises five variables: cancer type (including lung cancer), platelet count, hemoglobin level or use of erythropoiesis‐stimulating agents, leukocyte count, and body mass index (BMI). Patients are stratified into low (score of 0), intermediate (score of 1–2), and high‐risk (score ≥ 3) categories. The ONKOTEV score, a more recently validated model, includes a high Khorana score, presence of metastatic disease, vascular or lymphatic compression, and a history of VTE [32]. The risk of cardiovascular disease was assessed according to the COMPASS‐CAT (Computerized Registry of Patients with Solid Tumors for the Assessment of Venous Thromboembolism in Cancer—Clinical Assessment Tool) model for venous thrombosis risk. The factors considered include peripheral artery disease, ischemic stroke, coronary heart disease, hypertension, hyperlipidemia, diabetes mellitus, and obesity (BMI ≥ 30 kg/m^2^) [33].

In accordance with the regulations of the Clinical Research Ethics Committee of the First Affiliated Hospital, Zhejiang University School of Medicine (Approval Number: IIT20220496), this study was approved by the ethics committee, and the requirement for informed consent was waived.

### 2.3 Study Endpoints

The primary endpoint of the study was the association between TEs and oncogenic driver alterations, specifically *ALK*, *ROS1*, and *RET* rearrangements compared with *EGFR* mutations, during the peri‐diagnostic period (defined as 6 months before or after the diagnosis of advanced lung adenocarcinoma) in patients with advanced lung adenocarcinoma. This time frame was chosen to minimize potential confounding and capture early thrombotic events potentially related to tumor biology.

Secondary endpoints included: (1) the association between driver gene alterations and TEs over the entire follow‐up period; (2) the cumulative incidence of TEs during both the peri‐diagnostic period and the entire follow‐up period; (3) the identification of additional clinical factors independently associated with TE risks; and (4) OS in the entire cohort and in subgroups stratified by TE occurrence and driver gene status.

### 2.4 Statistical Analysis

Patient characteristics are presented as median (inter‐quartile range, IQR) for continuous variables and as frequencies for categorical variables. Baseline characteristics were compared using variance analysis, Kruskal‐Wallis test, Fisher's exact test, and the Mantel–Haenszel trend test. *p* values for pairwise comparisons within groups were adjusted using the Bonferroni method.

The cumulative incidence of TE was calculated from 1 year before cancer diagnosis to the time of TEs, death, or the last medical follow‐up record (November 1, 2021). The peri‐diagnostic period was defined as 6 months before and after the diagnosis of lung adenocarcinoma. The Fine‐Gray competitive risk regression model was used to evaluate the cumulative incidence of TEs [34], with deaths regarded as competing risks.

Univariate and multivariate analyses were performed using logistic regression analysis. We tested for collinearity using the variance inflation factor (VIF) and defined problematic collinearity as a VIF > 10 [35]. Variables with biological rationality and *p* < 0.05 in the univariate analysis results for the entire follow‐up or peri‐diagnostic period, as well as those relevant to TE occurrence from previous studies, were included in the multivariate analysis.

OS was analyzed using Kaplan–Meier curves, with OS compared between TE and non‐TE groups using the log‐rank test. The correlation between TE and OS was assessed using Cox regression, adjusted for sex, age, smoking history, TNM stage, and driver genes.

Statistical analyses were performed using IBM SPSS (25.0) software and R (3.6.1). Two‐sided tests were used, and *p* < 0.05 was considered statistically significant.

## 3 Results

### 3.1 Baseline Characteristics of Patients

**FIGURE 1:**
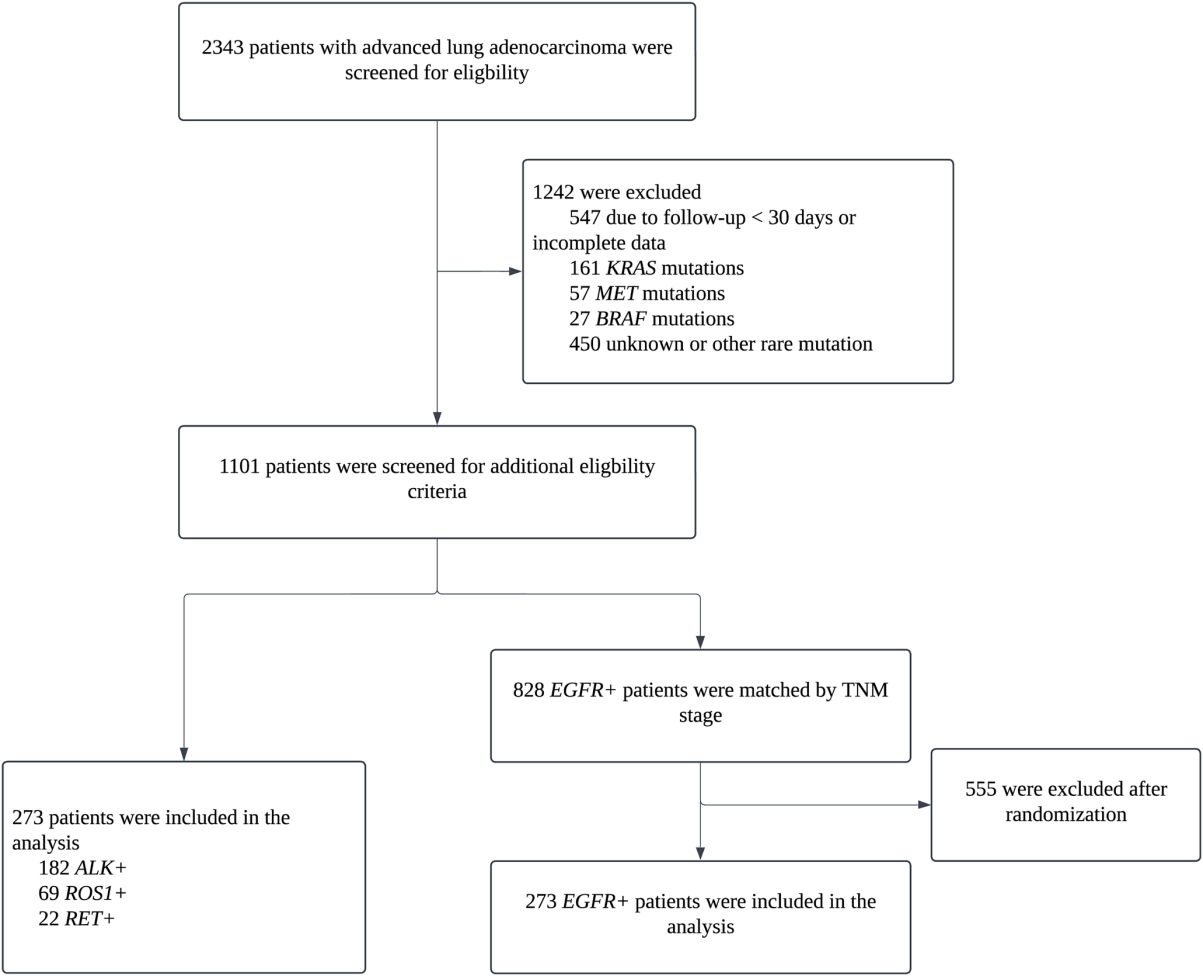
Study flow chart.

During the study, 2343 patients with advanced lung adenocarcinoma were diagnosed, including 1167 *EGFR*+, 327 *ALK*+, 98 *ROS1*+, and 56 *RET*+ patients. After screening, *EGFR*+ patients were stratified by TNM stage, and a subset was randomly selected (1:1) to match the number of *ALK*+, *ROS1*+, and *RET*+ patients (Figure 1). Ultimately, 182 *ALK*+, 69 *ROS1*+, 22 *RET*+, and 273 *EGFR*+ patients were included in this study. Baseline clinical characteristics are detailed in Table 1.

**TABLE 1:** Baseline demographics and clinical characteristics of patients with *ALK*, *ROS1*, *RET* rearrangements and *EGFR* mutations.

	*ALK*+ (*N* = 182)	*ROS1*+ (*N* = 69)	*RET*+ (*N* = 22)	*EGFR*+ (*N* = 273)	*p*
Sex (%)					0.327
Male	87 (47.8)	32 (46.4)	6 (27.3)	129 (47.3)	
Female	95 (52.2)	37 (53.6)	16 (72.7)	144 (52.7)	
Median age (range)	53 (21–81)	52 (26–82)	61 (37–89)	61 (27–85)	**< 0.001**
BMI	22.9 (21.2–24.5)	23.6 (21.6–25.9)	23.3 (21.7–24.7)	22.3 (20.3–24.8)	0.111
ECOG (%)					0.523
0–1	141 (77.5)	56 (81.2)	16 (72.7)	232 (85.0)	
2–4	41 (22.5)	13 (18.8)	6 (27.3)	41 (15.0)	
Smoking status (%)					0.866
Never	130 (71.4)	47 (68.1)	15 (68.2)	185 (67.8)	
Former/current	52 (28.6)	22 (31.9)	7 (31.8)	88 (32.2)	
Stage (%)					0.844
IIIB‐IIIC	29 (15.9)	12 (17.4)	3 (13.6)	44 (16.1)	
IVA	53 (29.1)	22 (31.9)	11 (50.0)	86 (31.5)	
IVB	100 (54.9)	35 (50.7)	8 (36.4)	143 (52.4)	
Number of comorbidities (%)					0.110
0–1	174 (95.6)	61 (88.4)	19 (86.4)	247 (90.5)	
≥ 2	8 (4.4)	8 (11.6)	3 (13.6)	26 (9.5)	
Khorana Score (%)					**0.005**
1–2	172 (94.5)	65 (94.2)	22 (100.0)	271 (99.3)	
≥ 3	10 (5.5)	4 (5.8)	0 (0.0)	2 (0.7)	
ONKOTEV Score (%)					**< 0.001**
0–1	107 (58.8)	37 (53.6)	16 (72.7)	215 (78.8)	
≥ 2	75 (41.2)	32 (46.4)	6 (27.3)	58 (21.2)	
Median follow‐up time (IQR)	19 (10–43)	28 (12–50)	19 (8–37)	23 (12–39)	0.103
TE (%)	29 (15.9)	24 (34.8)	7 (31.8)	36 (13.2)	**0.001**

*Note:* Bold values indicate statistically significant differences (*p* < 0.05).

Abbreviations: BMI, body mass index; ECOG, eastern cooperative oncology group performance status; IQR, inter‐quartile range.

The majority of patients with all these driver genes at advanced stages were female and non‐smokers. The age distribution among the four groups showed significant differences. Patients with *ALK* and *ROS1* rearrangements exhibited a similar age distribution, with patients in these groups being relatively younger (*ALK*+ vs. *EGFR*+, *p* < 0.001; *ALK*+ vs. *RET*+, *p* = 0.030; *ROS1*+ vs. *EGFR*+, *p* = 0.004).

There were significant differences in Khorana and ONKOTEV scores among the four groups (*p* = 0.005; *p* < 0.001). Specifically, patients with *ALK* rearrangements had significantly higher Khorana and ONKOTEV scores compared to those with *EGFR* mutations (*p* = 0.012; *p* = 0.016). No significant difference in follow‐up time was observed among the four groups. As shown in Table 1, the incidence of TEs was significantly higher in patients with *RET* and *ROS1* rearrangements compared to those with *EGFR* mutations (*p* = 0.024; *p* = 0.012).

### 3.2 Clinical and Therapeutic Characteristics Among Molecular Groups

The clinical characteristics of the four groups are summarized in Table 2. There were no significant differences in the distribution of primary tumor sites (*p* = 0.055). However, patients with *ALK* and *ROS1* rearrangements had significantly larger mediastinal lymph nodes at the time of diagnosis compared to those with *EGFR* mutations (both *p* < 0.001) and were more frequently staged with advanced nodal metastasis at diagnosis (both *p* < 0.001). In terms of metastasis patterns, patients with *ROS1* and *RET* fusions had a significantly higher incidence of distant lymph node metastases than patients with *EGFR* mutations (*ROS1*+ vs. *EGFR*+, 11.6% vs. 2.9%, *p* = 0.036; *RET*+ vs. *EGFR*+, 18.2% vs. 2.9%, *p* = 0.048). These findings suggest that *ALK*, *ROS1*, and *RET* rearrangements may be associated with a more aggressive lymphatic phenotype, potentially involving extensive nodal enlargement and dissemination. Such characteristics could lead to vascular or lymphatic compression, thereby increasing the risk of thromboembolic events through mechanical obstruction or endothelial activation.

**TABLE 2:** Clinical and treatment characteristics of patients with *ALK*, *ROS1*, *RET* rearrangements, and *EGFR* mutations.

	*ALK*+ (*N* = 182)	*ROS1*+ (*N* = 69)	*RET*+ (*N* = 22)	*EGFR*+ (*N* = 273)	*p*
Longest diameter of the primary lesion (cm) (Median, IQR)	3.1 (2.1–4.5)	3.3 (1.9–5.0)	2.8 (1.6–3.8)	3.4 (1.7–4.6)	**0.038**
Location of the primary lesion (%)					0.055
Peripheral	112 (61.5)	47 (68.1)	18 (81.8)	197 (72.2)	
Central/mediastinum	70 (38.5)	22 (31.9)	4 (18.2)	76 (27.8)	
Short diameter of mediastinal lymph nodes (cm) (Median, IQR)	1.4 (1.0–1.8)	1.3 (1.0–1.9)	1.2 (0.7–1.8)	1.0 (0.0–1.4)	**< 0.001**
*N* stage^a^ (%)					**< 0.001**
N0–2	72 (39.6)	16 (23.2)	8 (36.3)	164 (60.1)	
N3	110 (60.4)	53 (76.8)	14 (63.6)	109 (39.9)	
Serous cavity effusions (%)					
Pleural effusions	72 (39.6)	40 (58.0)	9 (40.9)	126 (46.2)	0.067
Pericardial effusions	28 (15.4)	12 (17.4)	2 (9.1)	22 (8.1)	**0.035**
Number of distant metastatic sites (%)					0.812
0	58 (31.9)	23 (33.3)	9 (40.9)	101 (37.0)	
1–2	102 (56.0)	40 (58.0)	11 (50.0)	141 (51.6)	
≥ 3	22 (12.1)	6 (8.7)	2 (9.1)	31 (11.4)	
Metastatic sites at diagnosis (%)					
Lung	46 (25.3)	22 (31.9)	7 (31.8)	99 (36.3)	0.107
Brain	43 (23.6)	17 (24.6)	1 (4.5)	40 (14.7)	**0.014**
Bone	61 (33.5)	16 (23.2)	4 (18.2)	113 (41.4)	**0.007**
Liver	25 (13.7)	5 (7.2)	2 (9.1)	15 (5.5)	**0.021**
Lymph nodes	13 (7.1)	8 (11.6)	4 (18.2)	8 (2.9)	**0.002**
Adrenal gland	13 (7.1)	5 (7.2)	3 (13.6)	18 (6.6)	0.595
Other	14 (7.7)	3 (4.3)	0 (0.0)	8 (2.9)	0.106
Systematic therapy (%)					
TKI (%)	164 (90.1)	56 (81.2)	7 (31.8)	266 (97.4)	**< 0.001**
Chemotherapy (%)	54 (29.7)	21 (30.4)	15 (68.2)	91 (33.3)	**0.006**
Immune therapy (%)	2 (1.1)	1 (1.4)	8 (36.4)	6 (2.2)	**< 0.001**
Antiangiogenic therapy (%)	23 (12.6)	8 (11.6)	10 (45.5)	41 (15.0)	**0.003**
Radiotherapy (%)	47 (25.8)	19 (27.5)	4 (18.2)	67 (24.5)	0.827
Untreated (%)	11 (6.0)	11 (15.9)	4 (18.2)	3 (1.1)	**< 0.001**

*Note:* Bold values indicate statistically significant differences (*p* < 0.05).

Abbreviations: IQR, inter‐quartile range; TKI, tyrosine kinase inhibitor.

^a^ The diagnosis of N stage was mainly based on radiographic findings.

Of the 546 patients, 29 patients had not received any treatment prior to TEs. Among treated patients, those with *EGFR* mutations had the highest proportion of using TKI (97.4%), significantly higher than that of the other three groups (*p* < 0.001). Patients with *RET* fusions had the highest proportion of receiving chemotherapy (68.2%), immunotherapy (36.4%) and anti‐angiogenic therapy (45.5%), significantly higher than that of the other three groups (*p* < 0.05).

Laboratory results are summarized in Table 3. CRP and carcinoembryonic antigen (CEA) levels were significantly higher in *ALK*+ patients compared with those with EGFR mutations (*p* = 0.012).

**TABLE 3:** Baseline laboratory results of patients with *ALK*, *ROS1*, *RET* rearrangements, and *EGFR* mutations.

	*ALK*+ (*N* = 182)	*ROS1*+ (*N* = 69)	*RET*+ (*N* = 22)	*EGFR+* (*N* = 273)	*p*
White blood cell, ×10^9^/L (%)					0.149
> 11	8 (4.4)	5 (7.2)	0 (0.0)	6 (2.2)	
≤ 11	171 (94.0)	64 (92.8)	22 (100.0)	267 (97.8)	
Hemoglobin, g/L (%)					**0.034**
< 100	15 (8.2)	6 (8.7)	1 (4.5)	8 (2.9)	
≥ 100	164 (90.1)	63 (91.3)	21 (95.5)	265 (97.1)	
Platelet, ×10^9^/L (%)					**0.031**
> 350	29 (15.9)	7 (10.1)	2 (9.1)	20 (7.3)	
≤ 350	150 (82.4)	62 (89.9)	20 (90.9)	253 (92.7)	
Aspartate aminotransferase, U/L (%)					0.474
> 40	14 (7.7)	3 (4.3)	1 (4.5)	12 (4.4)	
≤ 40	168 (92.3)	66 (95.7)	21 (95.5)	258 (94.5)	
Albumin, g/L (%)					1.000
< 35	21 (11.5)	6 (8.7)	1 (4.5)	34 (12.5)	
≥ 35	157 (86.3)	62 (89.9)	21 (95.5)	239 (87.5)	
Creatinine, μmol/L (%)					0.105
> Upper limit of normal creatinine^a^	7 (3.8)	2 (2.9)	2 (9.1)	23 (8.4)	
≤ Upper limit of normal creatinine	175 (96.2)	67 (97.1)	20 (90.9)	247 (90.5)	
Fibrinogen, g/L (%)					0.669
> 4	66 (36.3)	21 (30.4)	6 (27.3)	90 (33.0)	
≤ 4	112 (61.5)	48 (69.6)	16 (72.7)	181 (66.3)	
Prothrombin time, second (%)					**0.048**
> 13.5	14 (7.7)	4 (5.8)	0 (0.0)	7 (2.6)	
≤ 13.5	164 (90.1)	65 (94.2)	22 (100.0)	265 (97.1)	
D‐dimer, μg/L (%)					0.165
> 1500	60 (33.0)	28 (40.6)	6 (27.3)	78 (28.6)	
≤ 1500	106 (58.2)	40 (58.0)	16 (72.7)	191 (70.0)	
CRP, mg/L (%)					**0.025**
> 10	68 (37.4)	23 (33.3)	6 (27.3)	76 (27.8)	
≤ 10	90 (49.5)	43 (62.3)	14 (63.6)	190 (69.6)	
CEA, ng/mL (%)					**< 0.001**
> 5	107 (58.8)	32 (46.4)	16 (72.7)	201 (73.6)	
≤ 5	68 (37.4)	37 (53.6)	6 (27.3)	69 (25.3)	

*Note:* Bold values indicate statistically significant differences (*p* < 0.05).

Abbreviations: CRP, C‐reactive protein; CEA, carcinoembryonic antigen.

^a^ According to the reference range of creatinine provided by the Department of Clinical Laboratory, the upper limit of normal value is 111 μmol/L for men and 73 μmol/L for women.

### 3.3 Thromboembolic Events Among Molecular Groups

Among all included patients, a total of 69 patients experienced 101 TEs during the entire follow‐up period, comprising 71 cases of DVT (70.3%), 21 cases of PE (20.8%) and 9 cases of ATE (8.9%). Among these, 17 cases were recurrent TEs (16.8%), as detailed in Table 4. The incidence of ATE was significantly higher in patients with *RET* fusions than that in patients with *ALK* fusions (44.4% vs. 3.4%, *p* = 0.032).

**TABLE 4:** Thromboembolic events in patients with *ALK*, *ROS1*, *RET* rearrangements, and *EGFR* mutations.

	*ALK*+ (*N* = 29)	*ROS1*+ (*N* = 24)	*RET*+ (*N* = 7)	*EGFR*+ (*N* = 36)	*p*
Type (%)					
DVT	20 (69.0)	18 (75.1)	4 (44.4)	29 (74.4)	0.382
Upper extremity and neck	8 (27.6)	1 (4.2)	0 (0.0)	2 (5.1)	
Lower extremity and pelvis	12 (41.4)	16 (66.7)	4 (44.4)	26 (66.7)	
Upper and lower extremity	0 (0.0)	1 (4.2)	0 (0.0)	1 (2.6)	
PE	8 (27.6)	5 (20.8)	1 (11.1)	7 (17.9)	0.815
Segmental/subsegmental	0 (0.0)	2 (8.3)	0 (0.0)	2 (5.1)	
Above segmental	8 (27.6)	3 (12.5)	1 (11.1)	5 (12.8)	
ATE	1 (3.4)	1 (4.2)	4 (44.4)	3 (7.7)	**0.007**
Symptoms present (%)					0.987
Symptomatic	21 (72.4)	18 (75.0)	7 (77.8)	27 (69.2)	
Asymptomatic	8 (27.6)	6 (25.0)	2 (22.2)	12 (30.8)	
Recurrent TE (%)					0.309
With anticoagulant treatment	2 (6.9)	1 (4.2)	2 (22.2)	4 (10.3)	
Without anticoagulant treatment	2 (6.9)	1 (4.2)	1 (11.1)	4 (10.3)	
Therapeutic status at TE (%)					
Untreated	9 (31.0)	10 (41.7)	5 (55.6)	8 (20.5)	0.104
TKI	17 (58.6)	13 (54.2)	1 (11.1)	22 (56.4)	0.056
Chemotherapy	2 (6.9)	0 (0.0)	3 (33.3)	9 (23.1)	**0.009**
Others^a^	7 (24.1)	2 (8.3)	3 (33.3)	3 (7.7)	0.067
RECIST assessment at TE (%)					**0.061**
At diagnosis	11 (37.9)	11 (45.8)	4 (44.4)	8 (20.5)	
Disease progression	11 (37.9)	5 (20.8)	2 (22.2)	22 (56.4)	
Stable disease	6 (20.7)	5 (20.8)	3 (33.3)	4 (10.3)	
Partial response	1 (3.4)	3 (12.5)	0 (0.0)	5 (12.8)	
Median time of TE (days, IQR)	164 (6–464)	58 (11–194)	139 (−9–179)	315 (71–691)	0.177

*Note:* Bold values indicate statistically significant differences (*p* < 0.05).

Abbreviations: ATE, arterial thromboembolism; DVT, deep vein thrombosis; IQR, inter‐quartile range; PE, pulmonary embolism; RECIST, response evaluation criteria in solid tumors; TE, thromboembolic event; TKI, tyrosine kinase inhibitor.

^a^ Therapeutic states included antiangiogenic therapy, catheterization, post‐operation, radiotherapy, and immune therapy. There may be multiple therapeutic states when TE occurs. Multiple TE events were recorded as separate events.

**FIGURE 2:**
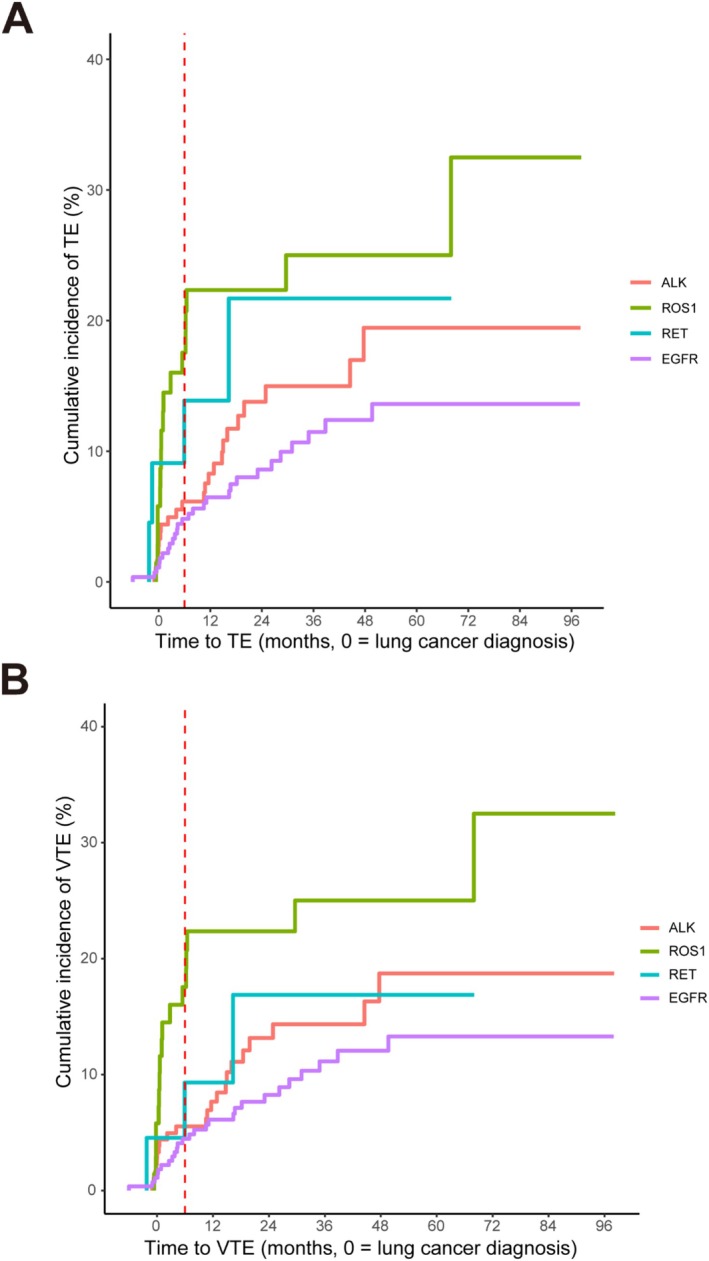
Cumulative incidence of thromboembolic events and venous thromboembolism in patients with *ALK*, *ROS1*, *RET* fusions and *EGFR* mutations. (A) Cumulative incidence of TEs in patients with different driver genes; (B) Cumulative incidence of VTE in patients with different driver genes. Only the first occurrence of TE or VTE in each case was recorded. The analysis was based on the Fine‐Gray competitive risk model, with deaths regarded as a competing event. The peri‐diagnostic period was defined as 6 months before and after the date of lung cancer diagnosis (time = 0). The red dashed line represents the 6‐month time point. TE, thromboembolic event; VTE, venous thromboembolism.

The cumulative incidence of TE is shown in Figure 2A. As for the peri‐diagnostic period (within 6 months before and after diagnosis), there is no significant difference between the four groups (*χ* ^2^ = 6.75, *p* = 0.080). The 6‐month cumulative incidence of TE was 17.5% ± 0.2%, 13.9% ± 0.6%, 6.2% ± 0.0%, and 4.8% ± 0.0% for patients with *ROS1*, *RET*, *ALK* fusions, and *EGFR* mutations, respectively. When considering the entire follow‐up period, the cumulative incidence of TEs among the four groups was significantly different (*χ* ^2^ = 11.27, *p* = 0.001). The overall cumulative incidence of TE was 32.5% ± 0.8%, 21.7% ± 1.1%, 19.5% ± 0.2%, and 13.6% ± 0.1% for patients with *ROS1*, *RET*, *ALK* fusions, and *EGFR* mutations, respectively. Among them, the cumulative incidence of TEs in patients with *ROS1* fusions was significantly higher than that in patients with *ALK* fusions and *EGFR* mutations (*χ* ^2^ = 4.14, *p* = 0.042; *χ* ^2^ = 10.65, *p* = 0.001). The incidence of TEs in patients with *ROS1* and *RET* fusions showed a rapid increase in the initial short term, while the incidence of TE in patients with *ALK* fusions and *EGFR* mutations gradually increased with the extension of follow‐up time. As for the cumulative incidence of VTE, there was a significant difference between the four molecular subgroups during the peri‐diagnostic period (*χ* ^2^ = 15.79, *p* = 0.001) as well as over the entire follow‐up period (*χ* ^2^ = 11.34, *p* = 0.010). Although ATE events were relatively rare, their cumulative incidence also differed significantly across the four groups during the peri‐diagnostic period (*χ* ^2^ = 28.24, *p* = 0.003) and over the entire follow‐up period (*χ* ^2^ = 51.85, *p* = 0.003). Notably, patients with *RET* fusions had the highest cumulative incidence of ATE, reaching 9.1% ± 0.4% in the peri‐diagnostic period and 13.9% ± 0.6% throughout the entire follow‐up.

Concerning the timing of TE, there was no significant difference in disease progression between groups when TE occurred (*p* = 0.061), as shown in Table 4. However, patients with *ALK*, *ROS1*, and *RET* fusions were more likely to develop TEs at diagnosis (*ALK*+ 37.9%; *ROS1*+ 45.8%; *RET*+ 44.4%), while those with *EGFR* mutations had a TE incidence of 56.4% during disease progression.

### 3.4 The Association Between Clinical Characteristics and Thromboembolic Events

The results of univariate analysis are presented in Table 5, encompassing all risk factors during the entire and peri‐diagnostic period (*p* < 0.05), along with risk factors considered to be associated with TEs in previous studies. Univariate analysis revealed that *ROS1* fusion was a significant risk factor for TEs in both the peri‐diagnostic period (OR = 3.51, 95% CI 1.68–7.31, *p* = 0.001) and the entire course (OR = 2.98, 95% CI 1.51–5.86, *p* = 0.002). During the peri‐diagnostic period, other independent risk factors for increased TE risk included age (OR = 1.04, 95% CI 1.01–1.07, *p* = 0.016), ONKOTEV score (OR = 2.22, 95% CI 1.15–4.28, *p* = 0.017), comorbidities, lymph node stage (OR = 1.60, 95% CI 1.08–2.36, *p* = 0.019), lymph node size (OR = 1.99, 95% CI 1.36–2.90, *p* < 0.001), and CRP level (OR = 3.48, 95% CI 1.77–6.82, *p* < 0.001). In the entire cohort, comorbidities, lymph node stage (OR = 1.29, 95% CI 1.00–1.66, *p* = 0.049), lymph node size (OR = 1.36, 95% CI 1.01–1.83, *p* = 0.040), and CRP level (OR = 2.58, 95% CI 1.20–5.56, *p* = 0.016) remained independent risk factors for increased TE risk.

**TABLE 5:** Univariate logistic analysis of the correlation between clinical characteristics and thromboembolic events.

	TE in the peri‐diagnostic period OR (95% CI)^a^	TE throughout the course OR (95% CI)
Driver gene		
*ALK*+ vs. *EGFR*+	0.77 (0.38–1.59), *p* = 0.482	1.32 (0.73–2.38), *p* = 0.360
*ROS1*+ vs. *EGFR*+	3.51 (1.68–7.31), ***p* = 0.001**	2.98 (1.51–5.86), ***p* = 0.002**
*RET*+ vs. *EGFR*+	2.14 (0.60–7.57), *p* = 0.238	2.02 (0.64–6.42), *p* = 0.231
Age	1.04 (1.01–1.07), ***p* = 0.016**	1.02 (1.00–1.04), *p* = 0.051
BMI (per 1 kg/m^2^ increase)	1.06 (0.96–1.17), *p* = 0.249	1.07 (0.99–1.16), *p* = 0.076
TNM stage	0.95 (0.61–1.46), *p* = 0.799	0.92 (0.66–1.29), *p* = 0.639
Khorana score (≥ 3 vs. 1–2)	0.86 (0.11–6.67), *p* = 0.884	0.44 (0.06–3.40), *p* = 0.433
ONKOTEV score (≥ 3 vs. ≤ 2)	2.22 (1.15–4.28), ***p* = 0.017**	1.14 (0.67–1.93), *p* = 0.629
Comorbidities		
Number of comorbidities	1.87 (1.26–2.75), ***p* = 0.002**	1.51 (1.09–2.09), ***p* = 0.012**
Risk of cardiovascular disease^b^	2.24 (1.15–4.35), ***p* = 0.017**	1.82 (1.09–3.03), ***p* = 0.022**
Hypertension	2.44 (1.26–4.71), ***p* = 0.008**	2.02 (1.21–3.39), ***p* = 0.008**
Diabetes	3.23 (1.25–8.38), ***p* = 0.016**	1.53 (0.61–3.85), *p* = 0.365
Metastatic characteristics at diagnosis		
Short diameter of mediastinal lymph nodes	1.99 (1.36–2.90), ***p* < 0.001**	1.36 (1.01–1.83), ***p* = 0.040**
N stage	1.60 (1.08–2.36), ***p* = 0.019**	1.29 (1.00–1.66), ***p* = 0.049**
Distant lymph node metastasis	2.52 (0.91–6.93), *p* = 0.074	1.89 (0.79–4.53), *p* = 0.154
Laboratory test at baseline		
D‐dimer (> 1500 μg/L vs. ≤ 1500 μg/L)	0.67 (0.28–1.57), *p* = 0.353	0.61 (0.32–1.19), *p* = 0.148
CRP (> 10 mg/L vs. ≤ 10 mg/L)	3.48 (1.77–6.82), ***p* < 0.001**	2.58 (1.20–5.56), ***p* = 0.016**
Firstline treatment		
TKI	1.10 (0.25–4.92), *p* = 0.897	0.49 (0.16–1.51), *p* = 0.217
Chemotherapy	1.95 (0.71–5.32), *p* = 0.192	1.39 (0.75–2.56), *p* = 0.291
Chemotherapy and anti‐angiogenic therapy	NA	0.56 (0.07–4.31), *p* = 0.578
Anti‐angiogenic therapy	NA	0.86 (0.37–1.99), *p* = 0.725
Radiotherapy	NA	1.12 (0.57–2.18), *p* = 0.745

*Note:* Bold values indicate statistically significant differences (*p* < 0.05).

Abbreviations: CRP, C‐reactive protein; NA, not applicable; TKI, Tyrosine kinase inhibitors.

^a^ The peri‐diagnostic period was defined as 6 months before and after the diagnosis of lung cancer.

^b^ Cardiovascular disease risk factors were defined according to the COMPASS‐CAT (Computerized Registry of Patients with Solid Tumors for the Assessment of Venous Thromboembolism in Cancer—Clinical Assessment Tool) model for venous thrombosis risk assessment, and included peripheral artery disease, ischemic stroke, coronary heart disease, hypertension, hyperlipidemia, diabetes mellitus, and obesity (BMI ≥ 30 kg/m^2^).

The results of multivariate analysis (as shown in Table 6) suggest that *ROS1* fusions (OR = 3.17, 95% CI 2.37–3.97, *p* = 0.005), the number of comorbidities (OR = 1.86, 95% CI 1.42–2.29, *p* = 0.005), mediastinal lymph node short axis (OR = 1.69, 95% CI 1.26–2.12, *p* = 0.017), and CRP levels (OR = 2.67, 95% CI 1.94–3.40, *p* = 0.008) were risk factors for peri‐diagnostic TEs. Additionally, *ROS1* fusions (OR = 2.45, 95% CI 1.81–3.09, *p* = 0.001) and hypertension (OR = 2.00, 95% CI 1.47–2.54, *p* = 0.110) were risk factors for the occurrence of TEs throughout the entire course.

**TABLE 6:** Univariate and multivariate logistic analysis of the correlation between clinical characteristics and thromboembolic event.

	TE in the peri‐diagnostic period OR (95% CI)		TE throughout the course OR (95% CI)	
	Univariate analysis	Multivariate analysis	Univariate analysis	Multivariate analysis
Driver genes				
*ALK*+ vs. *EGFR*+	0.77 (0.38–1.59), *p* = 0.482	/	1.32 (0.73–2.38), *p* = 0.360	/
*ROS1*+ vs. *EGFR*+	3.51 (1.68–7.31), ***p* = 0.001**	3.17 (2.37–3.97), ***p* = 0.005**	2.98 (1.51–5.86), ***p* = 0.002**	2.45 (1.81–3.09), ***p* = 0.006**
*RET*+ vs. *EGFR*+	2.14 (0.60–7.57), *p* = 0.238	/	2.02 (0.64–6.42), *p* = 0.231	/
Hypertension	2.44 (1.26–4.71), ***p* = 0.008**	/	2.02 (1.21–3.39), ***p* = 0.008**	2.00 (1.47–2.54), *p* = 0.110
Number of comorbidities	1.87 (1.26–2.75), ***p* = 0.002**	1.86 (1.42–2.29), ***p* = 0.005**	1.51 (1.09–2.09), ***p* = 0.012**	/
Short diameter of mediastinal lymph nodes/cm	1.99 (1.36–2.90), ***p* < 0.001**	1.69 (1.26–2.12), ***p* = 0.017**	1.36 (1.01–1.83), ***p* = 0.040**	/
CRP (> 10 mg/L vs. ≤ 10 mg/L)	3.48 (1.77–6.82), ***p* < 0.001**	2.67 (1.94–3.40), ***p* = 0.008**	2.58 (1.20–5.56), ***p* = 0.016**	/

*Note:* Bold values indicate statistically significant differences (*p* < 0.05).

Abbreviations: CRP, C‐reactive protein; TE, thromboembolic event; OR, odds ratio.

### 3.5 The Association Between Overall Survival and Thromboembolic Events

**FIGURE 3:**
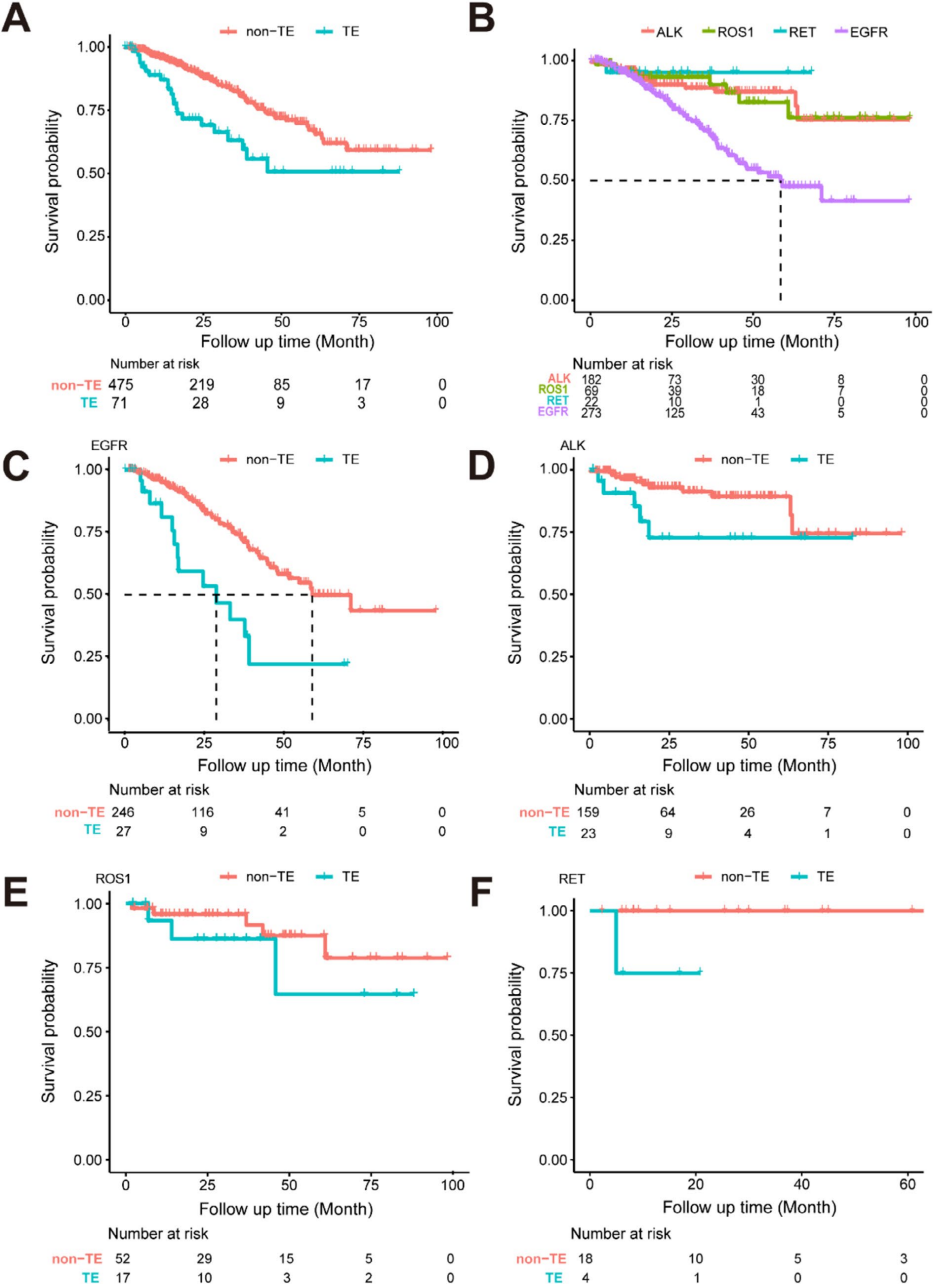
Kaplan–Meier survival curve of patients with or without TE. (A) Kaplan–Meier survival curve of all patients; (B) Kaplan–Meier survival curve of patients with different driver genes; (C–F) Kaplan–Meier survival curve of patients with *EGFR* mutations, *ALK* fusions, *ROS1* fusions, and *RET* fusions respectively. TE, thromboembolic event.

The occurrence of TEs significantly impacted the OS of patients, as shown in Figure 3. The risk of death was significantly increased in patients with TEs (HR = 3.92, 95% CI 2.33–6.59, *p* < 0.001) after adjusting for sex, age, smoking history, TNM stage, and driver genes.

Among patients with *EGFR* mutations, the risk of death in patients with TEs was approximately three times that of patients without TEs (HR = 2.99, 95% CI 1.20–7.46, *p* < 0.001), and the median survival of patients with and without TEs was 28.8 months (95% CI 16.7—NA) and 59.0 months (95% CI 48.2—NA), respectively. In patients with *ALK* and *ROS1* fusions, there was no significant difference in the risk of death between those with and without TEs (HR = 2.58, 95% CI 0.55–8.61, *p* = 0.064; HR = 2.12, 95% CI 0.40–11.27, *p* = 0.291). Among the patients with *RET* fusions, 18 patients did not develop TEs, and none died during follow‐up; four patients developed TEs, and one of them died.

## 4 Discussion

The correlation between TEs and oncogenic drivers in lung cancer is not well understood yet, necessitating further investigation, particularly concerning the elevated risks of TEs in patients with *ROS1* and *ALK* rearrangements. This study concentrates on assessing the risks of TEs in patients with *ROS1*, *ALK*, and *RET* rearrangements. Our findings indicate that among patients with advanced lung adenocarcinoma harboring driver genes, those with *ROS1* fusions exhibited the highest peri‐diagnostic and overall cumulative incidence of TEs. Multivariate analysis also revealed that *ROS1* fusions were significantly associated with an increased risk of TEs during both the follow‐up and peri‐diagnosis periods. Conversely, although patients with *ALK* fusions showed a slightly elevated overall cumulative incidence of TEs, their risk was not significantly higher compared to patients with *EGFR* mutations. Additionally, we observed a rapid increase in TE incidence among patients with *RET* rearrangements in the initial short term, with a significantly higher proportion of ATE compared to patients with *ALK* rearrangements.

In our cohort, the overall TE incidence was 15.9% in *ALK*+ patients and 13.2% in *EGFR*+ patients, with no significant difference in TE risks between these two groups. However, prior studies have reported conflicting results. For example, a population‐based Israeli study involving 4762 patients [36] reported increased TE risk in *ALK*+ compared with *ALK*‐wild type patients [10, 12, 21, 37]. In a U.S. cohort, the cumulative TE incidence was 45.3% for *ALK*+ and 21.2% for *EGFR*+ NSCLC over a median follow‐up of 33.1 months [38]. Similarly, Al‐Samkari et al. reported a significantly higher VTE incidence in *ALK*+ patients (42.7%) compared to *EGFR*+ patients (26.2%) [21]. In contrast, a Taiwanese study by H.Y. Wang et al. reported cumulative TE incidences of 7.38% in *ALK*+ and 6.1% in *EGFR*+ patients, while *ROS1*+ patients showed the highest incidence at 36% [39]. The discrepancies among these studies, including ours, may be attributed to variations in study design, patient populations, and underlying racial or genetic predispositions to thromboembolic risk.

Few studies have reported the real‐world incidence of TEs in patients with *RET* fusions. Limited data from clinical trials related to TKI therapy provide insights. In the phase 1/2 (ALL‐*RET*) study of alectinib, the TE incidence was 2.9% (one case of TE occurred in 34 patients) [40], while no TEs were reported in the phase II clinical trial of ponatinib for advanced NSCLC with *RET* fusions [41]. In contrast, a retrospective analysis reported a TE incidence as high as 48% in *RET*+ patients [30], highlighting significant variability in the reported incidence. In our cohort, the TE incidence for *RET*+ patients appeared to fall between those of *ROS1*+ and *ALK*+ patients. Similar to *ROS1*+, *RET*+ patients exhibited a rapid increase in TE incidence during the peri‐diagnostic period. Determining whether patients with *RET* fusions have an elevated risk of TE, particularly a predisposition to ATEs, requires further investigation in larger cohorts and prospective studies. In addition, the limited number of ATEs in this study constrained the scope of analysis. Further exploration into the potential association between ATE, driver genes, and the underlying biological mechanisms of VTE is warranted in future studies.

This study identified a significantly higher incidence of TEs in the patients with *ROS1* fusions, which may partly be explained by the relatively longer expected survival and follow‐up duration of *ROS1* patients compared to the general NSCLC population. To further explore this, we analyzed the peri‐diagnostic incidence of TEs across different driver gene subtypes. Notably, the risk of VTE in cancer patients is highest within the first 6 months after diagnosis [42, 43], a period less affected by confounding factors such as treatment regimens or follow‐up duration. In this study, the median time to TE onset in the *ROS1*+ and *RET*+ cohorts was less than 90 days. Furthermore, most TE events occurred around the time of diagnosis or disease progression, highlighting that TE is more likely to develop with a higher tumor burden. These findings suggest that the elevated peri‐diagnostic TE incidence in *ROS1*+ patients is likely driven by the biological effects of the oncogene itself. One proposed mechanism underlying the hypercoagulable state associated with *ROS1* rearrangements is the overproduction of mucin, a known thrombotic precursor [44, 45]. Additionally, *ROS1+* tumors are more prone to lymph node involvement, which has been linked in some studies to an increased risk of thrombosis [46].

There is a significant variability in the reported incidence of ATE among different driver genes. A recent study reported an elevated ATE incidence in *ROS1*+ NSCLC (7%, *N* = 193) [14]. However, another study reported only one case of ATE among 42 patients with *ROS1* fusions, yielding an incidence of 2% [47]. Contrasting these findings, our study did not reveal a significant increase in ATE incidence among patients with *ROS1* fusions, with only one ATE observed in a cohort of 69 *ROS1*+ patients (1.4%).

In this study, no significant association was observed between treatment regimens and the risk of TEs among patients with driver genes. The patients included in this study received TKI therapy as the main treatment regimen. A meta‐analysis that included 6 randomized controlled clinical trial studies showed that compared with platinum‐based chemotherapy, TKI treatment did not significantly increase the risk of thrombosis [48]. Additionally, in our study, 45.8% of TEs occurred in untreated patients with *ROS1* fusions, with 20.8% of TEs occurring at the time of disease progression. A prospective study [22] reported a VTE incidence of 41.6% in the *ROS1+* cohort, with 35.7% of VTE events occurring at the time of progression and 32.1% at the time of disease diagnosis, which is consistent with our findings, showing that TEs are more likely to occur when the disease is not well controlled. Therefore, for patients with *ROS1* fusions, initiating anti‐tumor therapy as soon as possible to reduce tumor burden may decrease the risk of TEs.

Regarding the association between TEs and OS, we found that only patients with *EGFR* mutations exhibited a significantly increased risk of death in the presence of TEs. In contrast, no significant difference in OS was observed between TE and non‐TE groups among patients with *ALK* and *ROS1* fusions. One possible explanation is that patients with *ALK* and *ROS1* fusions often achieve better responses to TKIs, leading to more favorable survival outcomes overall [49]. This therapeutic advantage may attenuate the impact of TEs on mortality in these subgroups. It is also worth noting that the lack of statistical significance in the *ALK* and *ROS1* cohorts could be related to limited sample sizes.

Our results reveal certain phenomena that require further validation through prospective studies, but they may have several implications for clinical practice. Firstly, it is imperative for patients with *ROS1* fusions to actively monitor signs and symptoms of TEs and undergo appropriate imaging examinations to rule out TE, especially during the peri‐diagnostic period. Prompt initiation of anti‐tumor therapy is recommended for patients with *ROS1* fusions during this period. Additionally, for patients at high risk of TEs (such as elevated CRP level, hypertension, large number of comorbidities and mediastinal lymphadenopathy), the necessity of primary thromboprophylaxis should be considered [50]. This study reaffirmed the elevated TE risk in patients with *ROS1* fusions, especially during the peri‐diagnostic period. It underscores the importance of incorporating driver genes as clinical variables when assessing TE risk, emphasizing the need for a comprehensive approach to risk prediction.

This study has several limitations. First, as a single‐center retrospective study conducted at a tertiary referral hospital with a Chinese patient population, it may introduce selection and referral bias, thereby restricting the generalizability of our findings to broader and more diverse populations. Second, the control group for TE analysis comprised patients with *EGFR* mutations, while the *EGFR*+ cohort was stratified based on TNM stage and matched randomly at a ratio of 1:1. Therefore, the distribution of each driver gene in this study may not accurately reflect real‐world proportions, potentially influencing some results. Third, although this study represents the largest cohort evaluating TEs in advanced lung adenocarcinoma with *RET* fusions, the cohort size remains relatively small due to the rarity of *RET* fusions in NSCLC, limiting the ability to detect significant differences among driver genes. Fourth, other driver genes beyond *ALK*, *ROS1*, *RET* fusions, and *EGFR* mutations were not evaluated. These subgroups were chosen for comparative analysis because *ALK* and *ROS1* fusions are well‐validated fusion genes in lung adenocarcinoma, whereas *EGFR* mutations are a commonly validated driver gene primarily receiving targeted therapy. Finally, information on anticoagulation uses and detailed treatment histories was incomplete, representing potential unmeasured confounders.

Despite these limitations, our study exhibits several strengths. First, we included a cohort of patients with *RET*‐rearranged lung cancer, a rare but therapeutically targetable subtype. We observed a potentially elevated incidence of TEs, particularly ATEs, in this group; however, this finding should be interpreted with caution due to the limited sample size and statistical power. Furthermore, our longitudinal follow‐up analysis of four driver gene cohorts reveals distinct temporal patterns and trends in TEs. Notably, *ROS1* and *RET* exhibit a higher TE incidence during the peri‐diagnostic period, while *ALK* and *EGFR* demonstrate a progressively increasing trend in TEs over time.

## 5 Conclusions

*ROS1* fusions exhibited the highest peri‐diagnostic and overall cumulative incidence of TEs among patients with advanced lung adenocarcinoma harboring driver genes. Multivariate analysis identified *ROS1* fusions as significantly associated with an increased risk of TEs during both the peri‐diagnosis period and follow‐up. Independent risk factors for TE development in the peri‐diagnostic period (within 6 months of diagnosis) included *ROS1* fusions, the number of comorbidities, mediastinal lymph node diameter, and CRP levels. Over the entire follow‐up period, *ROS1* fusions and hypertension were identified as independent risk factors for TEs.

## Author Contributions

All authors had full access to the data in the study and take responsibility for the integrity of the data and the accuracy of the data analysis. Conceptualization: X.Q., M.F., J.Z., J.Z. Methodology: X.Q., M.F., J.Z., J.Z. Investigation: X.Q., M.F., J.Z., J.J.C., C.C. Formal analysis: X.Q., M.F. Resources: J.Z., J.Z. Writing – original draft: X.Q., M.F. Writing – review and editing: X.Q., M.F., J.Z., J.Z. Visualization: X.Q., M.F. Supervision: J.Z., J.Z.

## Ethics Statement

In accordance with the regulations of the Clinical Research Ethics Committee of the First Affiliated Hospital, Zhejiang University School of Medicine (Approval Number: IIT20220496), this study was approved by the ethics committee, and the requirement for informed consent was waived.

## Conflicts of Interest

The authors declare that the research was conducted in the absence of any commercial or financial relationships that could be construed as a potential Conflicts of Interest.

## Data Availability

The clinical datasets generated and analyzed during this study are available from the corresponding author upon reasonable request, subject to approval from the institutional ethics committee.
